# Sweet’s Syndrome: A Case of Fever and Painful Erythematous Nodules Following a Viral-Like Illness

**DOI:** 10.7759/cureus.100701

**Published:** 2026-01-03

**Authors:** Maria Luis Santos, Inês Ferreira, Ussumane Embaló, Glória Alves, Jorge Cotter

**Affiliations:** 1 Internal Medicine Department, Unidade Local de Saúde Alto Ave, Guimarães, PRT

**Keywords:** corticosteroids, febrile neutrophilic dermatosis, neutrophilia, post-infectious inflammation, sweet’s syndrome

## Abstract

Sweet’s syndrome, or acute febrile neutrophilic dermatosis, is an uncommon inflammatory disorder characterized by the abrupt onset of fever, neutrophilia, and painful erythematous skin lesions. We report the case of a previously healthy woman who developed high fever, severe migratory arthralgias, and multiple tender erythematous nodules with pustular features following a recent viral-like illness. Laboratory evaluation revealed marked inflammatory activation with otherwise unremarkable hematological and biochemical parameters. A broad diagnostic investigation excluded infectious, autoimmune, endocrine, and neoplastic causes. Skin biopsy demonstrated a dense neutrophilic infiltrate without vasculitis, confirming Sweet’s syndrome. Systemic corticosteroid therapy led to rapid and sustained resolution of fever, arthralgias, and cutaneous lesions. This case illustrates the importance of early recognition and prompt biopsy in patients with acute febrile dermatoses to guide appropriate treatment and avoid unnecessary antimicrobial therapy.

## Introduction

Sweet’s syndrome, also known as acute febrile neutrophilic dermatosis, is characterized by the abrupt emergence of painful erythematous plaques or nodules, almost invariably accompanied by fever and peripheral neutrophilia. First described by Robert Douglas Sweet in 1964, the condition encompasses three major subtypes: classical (idiopathic or post-infectious), malignancy-associated, and drug-induced [1,2]. Histopathology typically reveals a dense neutrophilic infiltrate of the upper dermis in the absence of true leukocytoclastic vasculitis, a feature considered central to diagnosis.

Classical Sweet’s syndrome accounts for most cases and is often preceded by respiratory or gastrointestinal infection. The pathogenesis is thought to involve a dysregulated cytokine-mediated neutrophilic response triggered by infection, immune disturbance, or inflammatory activation, leading to dermal neutrophilic infiltration without true leukocytoclastic vasculitis, a histopathological feature that is diagnostically relevant [2-4]. Because its clinical manifestations overlap significantly with cellulitis, erythema nodosum, vasculitis, and other neutrophilic dermatoses, prompt recognition is needed to avoid unnecessary antimicrobial therapy and to initiate systemic corticosteroids, which typically induce a dramatic response.

We report a detailed case of classical Sweet’s syndrome with extensive systemic investigation, confirmed by histopathology, in a previously healthy woman presenting with fever, migratory arthralgias, and painful nodules following a viral-like illness. This case highlights the clinical complexity and the importance of structured etiological screening to exclude infectious, autoimmune, malignant, and drug-related triggers.

## Case presentation

A 43-year-old Caucasian woman presented with an eight-day history of fever, severe migratory arthralgias, and rapidly progressive painful cutaneous swellings. Her past medical history included vitiligo, with no active lesions at present, and benign breast fibrosis under stable radiological surveillance. She had no surgical history, no recent travel, no toxic exposures, no herbal supplements, and no drug allergies. She reported no recent direct contact with animals. 

She experienced an influenza-like illness in the preceding weeks, followed shortly afterward by her husband’s viral gastroenteritis; however, she remained entirely asymptomatic from a gastrointestinal standpoint. During this period, she completed a short course of amoxicillin-clavulanate prescribed for presumed bacterial sinusitis. She was otherwise well until she developed an abrupt high fever unresponsive to paracetamol and ibuprofen, associated with diffuse, severe, and disabling inflammatory arthralgias involving both large and small joints. The next day, she noted the appearance of painful erythematous swellings beginning on the right leg, thigh, and gluteal region, later progressing to the contralateral limb, upper limbs, trunk, and a single lesion on the face. She also reported mild tension-type headache and odynophagia but denied respiratory, gastrointestinal, or urinary symptoms.

She first sought emergency care, where she was discharged with etodolac and paracetamol. Her symptoms worsened, and she returned due to persistent fever, intense arthralgias limiting mobility, and progression of cutaneous lesions. On admission, she was febrile, hemodynamically stable, and fully oriented. Examination revealed multiple tender, indurated, erythematous nodules of varying size distributed across both lower limbs (more exuberant on the right), upper limbs, abdomen, and face. Several lesions exhibited pustular centers. No mucosal involvement was present. Cardiopulmonary, abdominal, and neurological examinations were otherwise unremarkable.

Initial laboratory studies showed a high-normal leukocyte count with neutrophil predominance, elevated C-reactive protein, preserved renal function, and normal electrolytes and liver enzymes (Table 1). Urinalysis revealed microscopic hematuria with no cylinders, leukocyturia, and epithelial cells, with the presence of yeast forms but negative nitrites. The respiratory viral PCR panel was negative. Chest radiography demonstrated an ill-defined reticular pattern in both lung bases, more evident on the right. Given the pustular appearance of some lesions, both blood and urine cultures were obtained, and empiric amoxicillin-clavulanate was initiated.

**Table 1:** Laboratory findings on admission and evolution. ALP, alkaline phosphatase; ALT, alanine aminotransferase; aPTT, activated partial thromboplastin time; AST, aspartate aminotransferase; CRP, C-reactive protein; ESR, erythrocyte sedimentation rate; GGT, gamma-glutamyl transferase; INR, international normalized ratio; LDH, lactate dehydrogenase; MCH, mean corpuscular hemoglobin; MCHC, mean corpuscular hemoglobin concentration; MCV, mean corpuscular volume; RBC, red blood cell; WBC, white blood cell; β-hCG, beta human chorionic gonadotropin

Parameter	Admission	Evolution	Reference Range
Hemoglobin	13.3 g/dL	11.7 g/dL	12-16 g/dL
Hematocrit	36.8 %	32.6 %	36-46 %
RBC count	4.39 ×10⁶/µL	3.85 ×10⁶/µL	4.0-5.2 ×10⁶/µL
MCV	83.8 fL	-	83-103 fL
MCH	30.3 pg	-	28-34 pg
MCHC	36.1 g/dL	-	32-36 g/dL
RDW	11.9 %	-	-
WBC count	9.5 ×10³/µL	7.8 ×10³/µL	4.8-10.8 ×10³/µL
Neutrophils	79.8 % (7.58 ×10³/µL)	63.2 %	1.8-7.7 ×10³/µL
Lymphocytes	9.4 % (0.89 ×10³/µL)	-	1.0-4.8 ×10³/µL
Monocytes	9.9 % (0.94 ×10³/µL)	-	0.12-0.80 ×10³/µL
Eosinophils	0.2 %	-	0.00-0.49 %
Basophils	0.3 %	-	0.0-0.1 %
Platelets	254 ×10³/µL	-	150-350 ×10³/µL
ESR	90 mm	-	0-12 mm
CRP	199.8 mg/L	16.0 mg/L	<3 mg/L
Procalcitonin	0.10 ng/mL	-	<0.1 / <0.5 ng/mL
Urea	24 mg/dL	-	15-39 mg/dL
Creatinine	0.59 mg/dL	0.56 mg/dL	0.55-1.02 mg/dL
Sodium	136 mEq/L	141 mEq/L	135-146 mEq/L
Potassium	4.42 mEq/L	3.70 mEq/L	3.5-5.1 mEq/L
Chloride	100 mEq/L	-	95-105 mEq/L
Calcium	9.4 mg/dL	-	8.3-10.6 mg/dL
Phosphorus	3.5 mg/dL	-	2.5-4.9 mg/dL
Magnesium	2.47 mg/dL	-	1.6-2.6 mg/dL
AST	25 U/L	15 U/L	12-40 U/L
ALT	11 U/L	13 U/L	7-40 U/L
GGT	13 U/L	-	0-38 U/L
ALP	39 U/L	-	46-116 U/L
LDH	350 U/L	195 U/L	120-246 U/L
Albumin	4.7 g/dL	-	3.4-5.0 g/dL
Total protein	8.1 g/dL	-	5.7-8.2 g/dL
Ferritin	368 ng/mL	-	10-291 ng/mL
Transferrin	159 mg/dL	-	250-380 mg/dL
β-hCG	<2 mIU/mL	-	<5 (non-pregnant)
INR	1.0	-	Normal
aPTT	28.3 s	-	Normal

The patient was admitted for further evaluation and management. Dermatology consultation raised suspicion for Sweet’s syndrome, and a punch biopsy of a right thigh lesion was performed. A comprehensive etiological evaluation was undertaken. Screening for HIV, HBV, and HCV was negative. Serology showed past exposure to rubella, varicella-zoster virus, cytomegalovirus, toxoplasmosis, and Epstein-Barr virus. Syphilis screening was negative. Blood and urine cultures were ultimately negative. CT of the thorax, abdomen, and pelvis detected no occult neoplasia or systemic involvement; only two small (8 mm) bilateral centrilobular ground-glass nodules were seen, interpreted as nonspecific and possibly post-infectious. Endocrine evaluation revealed normal thyroid function, with thyroid ultrasound showing a 6 mm mixed-echogenicity TI-RADS 2 nodule without suspicious features [5]. Breast ultrasound and mammography were BI-RADS 2 and unchanged from prior studies [6]. Bidirectional endoscopy revealed no significant abnormalities apart from mild gastric erythema; biopsies were taken and later returned benign. No evidence of extracutaneous Sweet’s syndrome was identified throughout the systemic evaluation.

Despite the initial use of antibiotics, the patient remained febrile and symptomatic; systemic corticosteroid therapy with prednisolone 1 mg/kg/day was initiated immediately after performing the skin biopsy. Within 48 hours, she experienced striking clinical improvement, with resolution of fever, rapid relief of arthralgias, and visible regression of the inflammatory component of the skin lesions. Laboratory follow-up performed at the same forty-eight-hour mark also demonstrated a clear analytical improvement, with a marked decline in inflammatory markers (Table 1). By the third day of corticosteroids, she resumed independent mobility and reported near-complete disappearance of joint pain. The cutaneous nodules progressively darkened and flattened, with the absence of new lesions.

The histopathology report confirmed Sweet’s syndrome, demonstrating a dense neutrophilic infiltrate in the upper dermis with papillary dermal oedema and absence of vasculitis. No microorganisms were identified. By the time of discharge, she was afebrile, pain-free, and her lesions were in clear resolution. She was discharged on a structured prednisolone taper of 10 mg per week and followed up in outpatient Internal Medicine. She remained under clinical follow-up with periodic reassessment, with no clinical relapse or laboratory abnormalities to date, with complete cutaneous remission and no recurrence, which is relevant given that relapse has been reported in a significant proportion of patients in published series [3,4].

**Figure 1:**
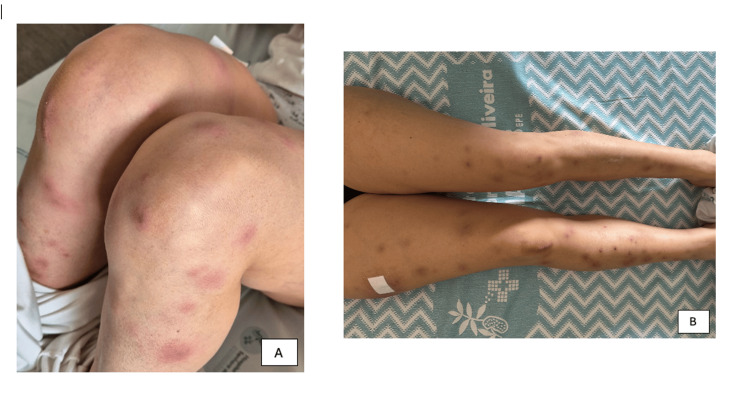
(A) Multiple tender, erythematous nodules with central pustules on the lower limbs at presentation. (B) Marked improvement 48 hours after initiation of systemic corticosteroid therapy, with flattening, darkening, and regression of the inflammatory component of the lesions.

The lesions, photographed on admission and after initiating corticosteroids, first showed large, sharply demarcated, raised erythematous nodules with central pustules and scattered confluence; in later stages, the lesions darkened and flattened, developing a violaceous hue consistent with resolving neutrophilic dermatosis (Figure 1).

## Discussion

Sweet’s syndrome is defined by the coexistence of characteristic skin lesions and requires correlation of clinical, histological, and laboratory features. According to the modified Su and Liu criteria and their subsequent refinements [1,2], diagnosis is established when both major criteria - typical painful erythematous plaques or nodules and characteristic neutrophilic dermal infiltrate without vasculitis - are present along with at least two minor criteria, such as fever, preceding infection or inflammatory trigger, elevated inflammatory markers or neutrophilia, and rapid response to corticosteroids. Although Sweet’s syndrome is classically associated with leukocytosis, it may also present with relative neutrophilia and markedly elevated inflammatory markers even in the absence of overt leukocytosis, as documented in published series [1,3,4,7].

This patient fulfilled all major and minor criteria. The abrupt onset of fever and painful nodules, the pattern of widespread distribution, the disabling migratory arthralgias, the markedly elevated inflammatory markers, and the prompt and dramatic steroid response provided strong clinical support. Histopathology confirmed the diagnosis by demonstrating the classic dense neutrophilic dermal infiltrate without vasculitis.

The extensive etiological work-up was essential to exclude other subtypes. There was no evidence of hematological disease, solid malignancy, active infection, or drug-induced mechanism. Amoxicillin-clavulanate, although associated with hypersensitivity reactions, began after the onset of systemic symptoms in this case and did not correlate with temporal criteria for drug-induced Sweet’s syndrome. Her preceding viral-like exposure, coupled with her husband’s recent gastroenteritis, suggests a post-infectious classical form, the most common subtype [3,4].

The lesions were initially interpreted as possibly infectious due to the presence of pustules, leading to empiric antibiotic therapy. However, the pustular variant of Sweet’s syndrome is well described and often misinterpreted as cellulitis or erysipelas [7]. The lack of biochemical or radiological evidence of bacterial infection and the absence of improvement with antibiotics further supported a non-infectious inflammatory process.

Corticosteroids remain the treatment of choice. Most patients respond within 48 to 72 hours, as seen here. Alternative agents such as colchicine, dapsone, or potassium iodide may be reserved for recurrent or refractory disease [8]. The risk of relapse is reported in up to one-third of patients, especially in the presence of persistent triggers or insufficient tapering. This patient demonstrated an excellent response with no recurrence to date.

This case underscores the importance of recognizing Sweet’s syndrome even when presentation includes atypical features such as pustules or multifocal distribution. It also highlights the need for broad systematic screening, particularly to exclude malignant and autoimmune associations, given that dermatological lesions may precede systemic disease by months, which emphasizes the importance of maintaining close follow-up. Relapse has been reported in up to one-third of patients in the literature [3,4], which reinforces the importance of continued clinical follow-up, although our patient has shown sustained remission to date.

## Conclusions

Sweet’s syndrome should be considered in patients presenting with abrupt fever, painful erythematous nodules, and elevated inflammatory markers, particularly when there is recent infection and poor response to antibiotics. Histopathology remains the diagnostic cornerstone. Systemic corticosteroids provide rapid and profound improvement. This case demonstrates a classical post-infectious presentation with a complete clinical recovery following prompt recognition and therapy, emphasizing the value of early dermatological assessment and structured etiological investigation.

## References

[1] Cohen PR, Kurzrock R (2003). Sweet's syndrome revisited: a review of disease concepts. Int J Dermatol.

[2] Su WP, Liu HN (1986). Diagnostic criteria for Sweet's syndrome. Cutis.

[3] Villarreal-Villarreal CD, Ocampo-Candiani J, Villarreal-Martínez A (2016). Sweet syndrome: a review and update. Actas Dermosifiliogr.

[4] Cohen PR (2007). Sweet's syndrome - a comprehensive review of an acute febrile neutrophilic dermatosis. Orphanet J Rare Dis.

[5] Tessler FN, Middleton WD, Grant EG (2017). ACR Thyroid Imaging, Reporting and Data System (TI-RADS): White Paper of the ACR TI-RADS Committee. J Am Coll Radiol.

[6] American College of Radiology (2022). American College of Radiology. ACR BI-RADS® Atlas: Breast Imaging Reporting and Data SystemReston, VA: American College of Radiology; 2022. Available from. ACR BI-RADS® Atlas: Breast Imaging Reporting and Data System.

[7] Kemmett D, Hunter JAA (1990). Sweet’s syndrome: a clinicopathologic review of twenty-nine cases. J Am Acad Dermatol.

[8] Walker DC, Cohen PR (1996). Trimethoprim-sulfamethoxazole-associated acute febrile neutrophilic dermatosis: case report and review of drug-induced Sweet's syndrome. J Am Acad Dermatol.

